# Deadwood effects on dissolved organic carbon in forest soils depend on bedrock type, tree species, and microclimate

**DOI:** 10.1038/s41598-026-50174-1

**Published:** 2026-04-28

**Authors:** Lisa Rubin, Rebecca Nowack, Friederike Lang, Peter Stiasny, Heike Puhlmann

**Affiliations:** 1 Department of Soil and Environment, Forest Research Institute Baden-Württemberg, Freiburg, Germany; 2 Department of Biometry and Informatics, Forest Research Institute Baden-Württemberg, Freiburg, Germany; 3 Chair of Soil Ecology, University of Freiburg, Freiburg, Germany

**Keywords:** Deadwood, Dissolved organic carbon, European beech, Norway spruce, Microclimate, Bedrock type, Biogeochemistry, Ecology, Ecology, Environmental sciences

## Abstract

Deadwood plays an important role in the forest carbon cycle by supplying dissolved organic carbon to the underlying soils. Yet, the extent to which this effect varies across different site conditions remains insufficiently understood. We monitored dissolved organic carbon at multiple soil depths beneath European beech and Norway spruce logs at sites with contrasting bedrock types over 2.5 years, comparing them with adjacent control plots. Concentrations were consistently elevated beneath deadwood, but the magnitude of the increase varied with bedrock type, tree species, and depth, and was influenced by soil temperature and moisture. Differences between deadwood and control were minor beneath the forest floor but pronounced in the upper mineral soil (15 cm), with the strongest increase (+ 90%) observed at the silicate site. When separating by tree species, this increase was driven by beech deadwood, while spruce showed little effect. At the silicate site, concentrations declined markedly between 15 and 30 cm, suggesting enhanced retention or processing in deeper layers. The effect of soil moisture on DOC was similar between deadwood and control plots, but temperature effects differed. These results indicate that the contribution of deadwood to soil carbon inputs is context-dependent, varying with tree species and soil properties.

**Supplementary Information:**

The online version contains supplementary material available at 10.1038/s41598-026-50174-1.

## Introduction

Deadwood plays an important role in biodiversity conservation^1–3^, especially in managed forests^4^. Increasing deadwood stocks has therefore become an important management objective and is already being promoted through deadwood conservation concepts^4^. In addition to its benefits for biodiversity, deadwood may also contribute to carbon (C) sequestration in forest soils^1,5,6^. To better understand the role of deadwood in the forest C cycle, it is important to identify and quantify the pathways of C released during wood decomposition^7^, as the fate of released C and the factors controlling it are not entirely clear^8^. Carbon is either released to the atmosphere as carbon dioxide (CO_2_) via respiration^9–11^ or transferred to the soil as particulate organic carbon (POC) or dissolved organic carbon (DOC)^12–14^. The leaching of DOC from deadwood into the mineral soil^1^, particularly into deeper soil layers^15^, may therefore play a key role in the formation of C stocks in forest soils, as deadwood represents a long-term DOC source^16^.

Downed deadwood logs decompose more rapidly than standing deadwood because of a higher moisture content^17^ and contact with the soil provides easier access for decomposers such as fungi, bacteria, and invertebrates. Breakdown and nutrient release are therefore often greater for logs directly connected to the soil surface^18^. Higher DOC concentrations indicate a greater availability of easily accessible C for microorganisms^19,20^, likely enhancing microbial activity and diversity beneath deadwood^8,19,21–23^. Increased microbial activity may, in turn, promote DOC release from the organic layer^20^ and the mineral soil^8,21,24,25^.

Several studies have reported higher DOC concentrations in soil under deadwood compared to control sites without deadwood^12,14,26–28^. The amount of DOC released from deadwood varies considerably among study sites^14,27,29^ and even among logs within a site^12^. DOC concentrations also vary with tree species^14^, with higher concentrations under broadleaved compared to coniferous species^30,31^. These differences are likely linked to the type of wood-decaying fungi: broadleaved species are typically decomposed by white-rot fungi, which efficiently break down lignin, whereas coniferous species are mainly decomposed by brown-rot fungi, which leave lignin largely intact^32^. As a result, coniferous species generally decompose more slowly and influence the soil at a slower pace^33^.

Beyond tree species, additional factors influence how deadwood affects DOC. For example, differences in deadwood effects on water extractable organic carbon (a proxy for DOC) were detectable only in soils on silicate bedrock compared to calcareous substrates^6^, indicating that substrate chemistry interacts with species-driven decomposition processes to shape DOC dynamics. In addition to these species- and substrate-specific effects, the degree of decomposition itself strongly affects DOC release. Deadwood can already act as a source of C via the dissolved phase in early decomposition stages^14^. Hafner, et al.^27^ even found that decomposition stage, rather than species, primarily determines DOC leaching rates. Spears, et al.^34^ and Spears and Lajtha^26^ observed peak DOC concentrations or fluxes at medium decomposition stages of coniferous deadwood. In contrast, Gonzalez-Polo, et al.^19^ reported highest DOC concentrations under broadleaved deadwood in the most advanced decay class.

Beyond species, substrate, and decomposition stage, microclimatic conditions in the immediate vicinity of deadwood further influence DOC dynamics. Downed deadwood logs can create a microclimate with increased soil moisture^35,36^, which is particularly important for ecosystem functions during dry periods. This effect is likely enhanced at advanced stages of decomposition, as water storage capacity increases with decay level^35^. Furthermore, deadwood can delay the drying of the underlying soil^37^, most likely by reducing evaporation. However, some studies found lower soil moisture beneath deadwood^38,39^. Deadwood may also lower soil temperatures during summer by intercepting solar radiation and acting as an insulator buffering soil temperature^38,40^.

In this study, we investigated the effects of deadwood on DOC in soil water across contrasting tree species, substrate types, and microclimatic conditions. Soil water was sampled beneath European beech (*Fagus sylvatica* L.) and Norway spruce (*Picea abies* (L.) H. Karst) deadwood, as well as at adjacent control sites without visible deadwood. Soil moisture and temperature, key parameters for decomposition, were recorded throughout the study.

We hypothesized that (i) the increase in DOC concentrations beneath deadwood is greater on silicate than on calcareous soils, (ii) DOC concentrations beneath beech deadwood are higher than beneath spruce deadwood under comparable site conditions, (iii) DOC concentrations beneath deadwood are influenced by a deadwood-controlled microclimate.

## Materials and methods

### Study sites

The study sites were selected to represent contrasting geological settings, including a silicate and a calcareous site, in order to capture differences in soil formation and biogeochemical processes related to parent material. The tree species comparison was conducted at the silicate site (gneiss) to ensure homogeneous substrate conditions. At the calcareous site (Jurassic limestone), two adjacent slopes with contrasting exposition (northeast vs. southwest) were selected to assess microclimatic variation while keeping substrate conditions comparable.

The silicate site is located in a managed mixed forest near Waldkirch in the western Black Forest, Germany (48°5’56’’N, 7°56’13’’E). The calcareous sites are located near Tuttlingen in the southwestern Swabian Alb, Germany, on two neighboring slopes with northeast (47°58’40’’N, 8°44’51’’E) and southwest exposition (47°58’58’’N, 8°45’7’’E), both dominated by European beech. Study site characteristics are presented in Table 1. Sampling was conducted with permission from the municipal foresters of Waldkirch and Tuttlingen.

**Table 1:** Study site characteristics including soil (according to IUSS Working Group WRB^41^, climate (mean annual precipitation (MAP) and mean annual temperature (MAT) (reference period 1991–2023, Deutscher Wetterdienst (DWD)), forest stand composition (visually estimated proportion of stand area (%)), and estimated stand age.

Study site	Reference Soil Group	Texture class	Humus form	Elevation (m a.s.l.)	Slope (°)	MAP (mm)	MAT (°C)	Forest stand composition	Stand age (years)
Waldkirch	Dystric Cambisol	Sandy loam	Mull	415	7	1100	10.4	50% *Fagus sylvatica*, 30% *Abies alba*, 10% *Pseudotsuga menziesii*, 10% *Picea abies*	95
Tuttlingen Northeast	Rendzic Leptosol	Silty clay	Mull	820	27	933	8.2	100% *Fagus sylvatica*	90
Tuttlingen Southwest	Rendzic Leptosol	Silty loam (topsoil), Silty clay (subsoil)	Mull	760	26	907	8.3	80% *Fagus sylvatica*, 20% *Acer platanoides*	100

### Sampling design

All sampled downed deadwood logs were already present in the stand and were not felled or placed for this study. At the Waldkirch site, six logs were selected: three of beech and three of spruce. At the Tuttlingen sites, only beech logs were selected, three on the northeast-facing slope and three on the southwest-facing slope. Log diameters ranged from 11 to 30.75 cm and lengths from 2.55 to 11.3 m. Decay classes were determined using the pocket-knife method described by Lachat, et al.^42^, which assigns deadwood to five decomposition stages based on wood resistance, corresponding to the five-class system of Maser, et al.^43^, ranging from recently deadwood (class 1) to highly decomposed wood (class 5). This approach provides a robust and widely used assessment of decomposition stage. However, it reflects the current decay status rather than time since tree death, which may vary among logs within the same class depending on species, log characteristics (e.g. diameter), and environmental conditions. Characteristics of the sampled logs are presented in Supplementary Table S1 online. The sampled logs differed in volume and slope position (parallel to slope or downslope), while decay stage was kept in a similar range (classes 3–4). All logs were in full contact with the forest floor. Each sampled deadwood log (*deadwood*) had a corresponding control plot (*control*) located within 5 m. Although past deadwood influence at the control plots cannot be excluded, no deadwood was present at installation and none was added during the study period.

### Soil solution sampling and field measurements

From September to November 2021, ceramic suction cups (5 cm length) were installed along the deadwood logs at a distance of approximately 5 cm from the stems and in the associated control plots. Horizontal suction cups were installed directly beneath the forest floor, while vertical suction cups were installed at depths of 15 cm and 30 cm in the mineral soil. To avoid moving the log or shielding the suction cups, they were installed as close as possible to the log but not directly underneath. For logs parallel to the slope, suction cups were positioned downslope to account for water movement; otherwise, they were distributed on both sides of the log. At each depth, three replicate suction cups were installed per log and control plot, with at least 20 cm distance between them. Samples collected from the suction cups at each depth were combined into a mixed sample.

Soil water was extracted using suctions pumps (SP 600 EC-VD, Schweizer Precision GmbH, Essen, Germany) at a negative pressure of -0.6 hPa. An automatic timer started the pumps six times per day for three minutes. Samples of the first few months after installation were discarded to allow the suction cups to settle. Thereafter, soil water samples were collected every four weeks.

ML3 Theta sensors (Delta-T Devices, Cambridge, UK) were installed at 15 cm depth to measure volumetric soil water content (SWC) and soil temperature. Two sensors were installed at each log and control plot to better capture spatial variability within the topsoil. Sensors were installed either below the logs (for slope-parallel stems) or laterally to the logs (for downslope-oriented stems), which may have altered log-induced microclimatic effects and contributed to variability in the measurements. All parameters were recorded every 30 min using a data logger (Delta-T Devices, Cambridge, UK). Soil temperature and water content measured at 15 cm were used as proxies for topsoil conditions, as no sensors were installed at other depths due to logistical constraints.

### Laboratory analysis

From 11 January 2022 until 30 September 2024, a total of 1,722 soil water samples were collected and analyzed. Samples were stored unfiltered at 5.8 °C (max. 24 h) until they were analyzed in the laboratory. The soil water was filtered through a cellulose acetate filter (0.45 μm, Sartorius, Goettingen, Germany). Turbid samples from beneath the forest floor were additionally pre-filtered using a glass microfiber filter (Whatman, Maidstone, United Kingdom). DOC concentrations were determined by continuous flow analysis (Auto Analyzer, Skalar Analytical B.V., Breda, Netherlands).

### Data analysis and statistics

#### Data preparation

All data preparation and analyses were conducted using the open-source software R version 4.5.0^44^. To ensure equal numbers of observations, only paired samples from both treatments at the same date and depth were included. Values below the limit of quantification (LOQ) were set to the LOQ, while values between the limit of detection (LOD) and the LOQ were set to the LOD following the recommendations of Helsel^45^. This approach retains observations below analytical limits while preserving their relative ranking in subsequent analyses. Outliers were initially identified through visual inspection using boxplots (values outside 1.5 x interquartile range). These observations were checked, and extremely high values attributable to sample contamination were removed from the dataset.

#### Calculation of relative available water capacity

Relative available water capacity (rAWC, %), hereafter referred to as soil moisture, was used to standardize SWC across study sites and depth to ensure comparability. Measured volumetric SWC (cm^3^ cm^− 3^) was aggregated to daily averages for each sensor. Then local maxima in SWC time series were filtered to estimate field capacity (FC in cm^3^ cm^− 3^), defined as the water content retained by the soil against gravity over a defined period (3 days). Maxima were first detected using the second derivative and subsequently filtered to remove values below the 85th percentile or occurring in close temporal proximity. Finally, decreasing moisture levels after each maximum were verified to ensure only relevant maxima were used to estimate FC. Permanent wilting point (PWP in cm^3^ cm^− 3^) was derived from the daily time series as the minimum value over the entire measurement period. Relative available water capacity (rAWC %) (Eq. 1) was calculated as follows:

1 $$\:rAWC\:\left(\%\right)=\frac{measured\:SWC\:\left({cm}^{3}{cm}^{-3}\right)-PWP\:\left({cm}^{3}{cm}^{-3}\right)}{FC\:\left({cm}^{3}{cm}^{-3}\right)-PWP\:\left({cm}^{3}{cm}^{-3}\right)}*100$$

### Statistical analysis

Linear mixed-effect models (LMEs) were used to identify factors influencing DOC concentrations while accounting for repeated measurements and the nested structure of the data. DOC concentrations were log-transformed to meet model assumptions, and residuals were inspected to ensure normality and homogeneity of variance. Separate models were fitted to analyze the effects of bedrock type and tree species, each including interactions with treatment (deadwood vs. control) and depth. Site (bedrock type model) or Tree ID (tree species model) was included as a random effect to account for repeated measurements and hierarchical data structure. For the tree species model, only data from the Waldkirch site, where both tree species were present, were used. Post-hoc comparisons of estimated marginal means were conducted to examine differences between factor levels.

To assess the effects of soil temperature and moisture on DOC concentrations, LMEs including interactions with treatment and depth were fitted. Soil temperature and moisture (15 cm) were represented as the mean values across each four-week period prior to DOC sampling. In addition, a separate LME was used to evaluate the effects of deadwood presence on soil temperature and moisture. The influence of continuous variables on DOC was assessed by estimating the slopes of their relationships with DOC. Differences in these slopes between treatments were used to determine whether temperature and moisture effects varied with the presence of deadwood. We considered results statistically significant at three levels: highly significant (*p* < = 0.001 ***), significant (*p* < = 0.05 **) and marginally significant (*p* < = 0.1 *). Graphical presentation was generated using the *ggplot2* package^46^.

## Results

### Effect of deadwood on DOC concentrations depending on bedrock type

**Fig. 1:**
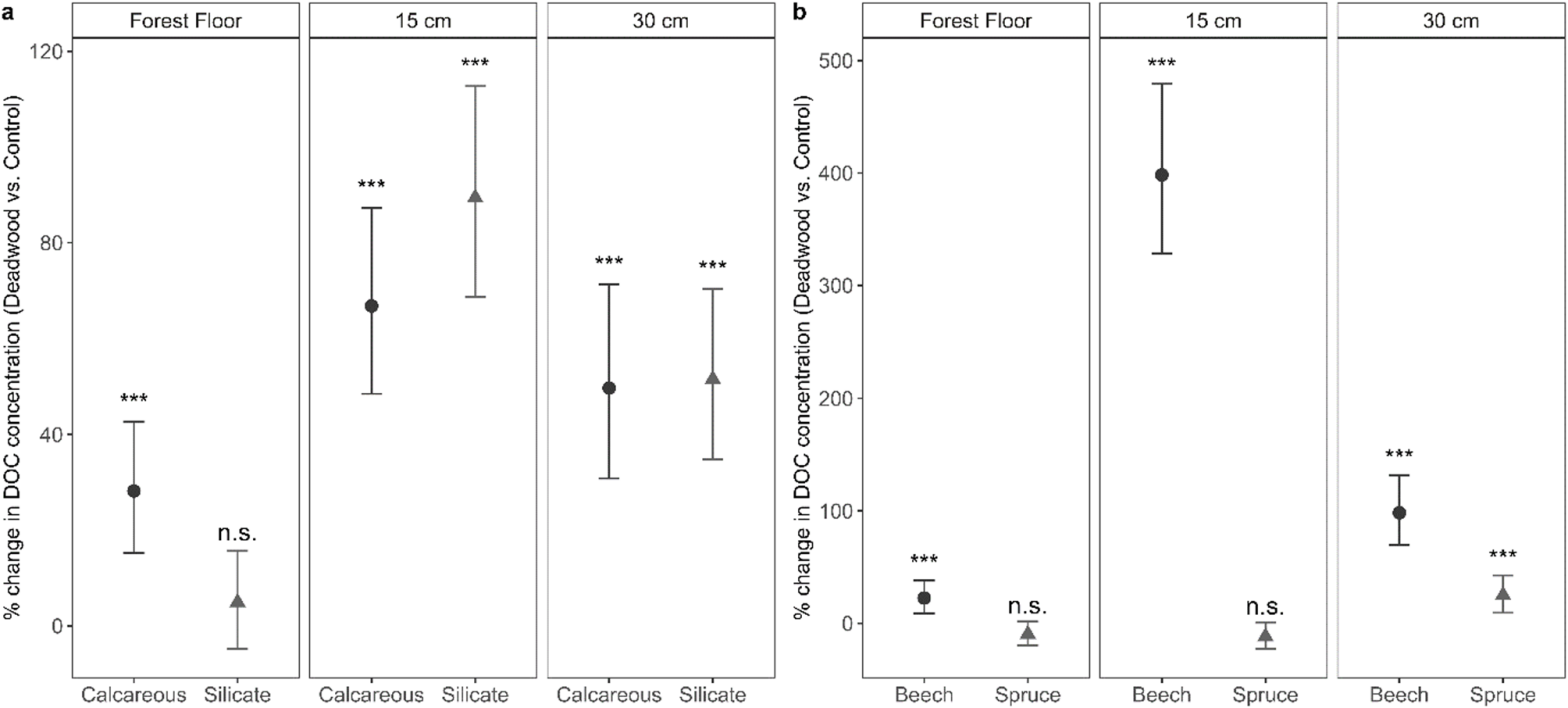
Depth-specific changes in DOC concentrations under deadwood relative to control. **a** Percentage change in DOC concentrations between deadwood and control treatments for calcareous and silicate soils across different soil depths. **b** Percentage change in DOC concentrations between deadwood and control treatments for beech and spruce across different soil depths. Symbols represent model-derived mean percentage changes, and error bars indicate 95% confidence intervals. Asterisks above the error bars denote statistically significant differences between deadwood and control (n.s. = not significant).

The linear mixed-effect model showed that deadwood generally increased DOC concentrations in the soil solution across depths and bedrock types (estimate = 0.25, *p* < 0.001; Supplementary Table S2). In addition, DOC concentrations decreased consistently with increasing depth (15 cm: estimate = −1.36, *p* < 0.001; 30 cm: estimate = -1.60, *p* < 0.001; Fig. 1a, Supplementary Table S2), but the presence of deadwood reduced this effect, indicating that DOC concentrations beneath deadwood are decreasing less compared to control plots.

The effect of deadwood varied also with bedrock type. Overall, the increase in DOC was slightly smaller at the silicate site (estimate = -0.20, *p* = 0.008). Independent of treatment (Deadwood/Control), DOC concentrations declined more strongly with depth at the silicate site compared to the calcareous site (15 cm: estimate = −0.62, *p* < 0.001; 30 cm: estimate = −0.92, *p* < 0.001).

Importantly, the three-way interaction between deadwood presence, bedrock type, and depth was significant, indicating that the effect of deadwood differs depending on both soil depth and bedrock. To further interpret this interaction, estimated marginal means (Supplementary Fig. S1) and percentage change in DOC under deadwood relative to the control were examined (Fig. 1a). These show that at 15 cm depth, the increase in DOC was greater at the silicate site (+ 90%), whereas at 30 cm depth, the increase was similar between silicate and calcareous site (+ 50%). Overall, differences between bedrock types diminished with depth.

### Effect of deadwood on DOC concentrations depending on tree species

The effect of deadwood differed strongly between tree species. The increase in DOC concentration was significantly smaller under spruce deadwood (estimate = -0.33, *p* < 0.001; Supplementary Table S3). In addition, the species-specific response varied with soil depth. At 15 cm, the deadwood effect differed strongly between species (three-way interaction, estimate = -0.64, *p* < 0.001; Supplementary Table S3), primarily driven by markedly higher values under beech compared to spruce.

To further examine these interactions, model-derived estimated marginal means (Supplementary Fig. S2) and percentage changes in DOC under deadwood relative to control (Fig. 1b) were analyzed. The increase in DOC concentrations was higher under beech deadwood compared to control, with the most pronounced difference at 15 cm depth. Significant increases in DOC concentrations occurred primarily beneath beech deadwood (+ 400% at 15 cm, + 100% at 30 cm). In contrast, under spruce, DOC declined relative to control directly beneath the forest floor and at 15 cm depth by 10 and 12%, respectively. However, this decline was not significant. Species-specific differences were most pronounced at intermediate soil depth and diminished both towards the forest floor and at 30 cm depth.

### Deadwood influence on soil temperature and soil moisture

Linear mixed-effect models were used to test the effect of deadwood presence on soil temperature and moisture. Deadwood had a significant but minor negative effect on soil temperature (estimate = -0.11, *p* = 0.090) (Table 2, Supplementary Fig. S3) and a stronger negative effect on soil moisture (estimate = -2.17, *p* < 0.001) (Table 2, Supplementary Fig. S3). Overall, these findings suggest that microclimatic conditions beneath deadwood differ from those at control plots, exhibiting slightly cooler and drier conditions, although effect sizes were relatively small.

**Table 2:** Results of the linear mixed-effects model testing the effect of deadwood on soil temperature and soil moisture.

Predictor	Estimate	Std. Error	95% CI	p-value
Soil Temperature				
Intercept	11.30	0.72	9.88–12.71	**< 0.001**
Treatment [Deadwood]	−0.11	0.07	-0.24–0.02	**0.090**
Soil Moisture				
Intercept	69.40	2.62	64.26–74.75	**< 0.001**
Treatment [Deadwood]	−2.17	0.50	−3.15 – −1.19	**< 0.001**

Estimates, standard errors (SE), 95% confidence intervals (CI), and p-values are shown. P-values were calculated using the Satterthwaite approximation.

### Microclimatic effects on DOC

LMEs testing the interacting effects of soil temperature and soil moisture with deadwood presence across soil depths showed a significant positive effect of soil temperature on DOC concentrations (estimate = 0.03, *p* < 0.001), while soil moisture showed no overall main effect (estimate = 0.00, *p* = 0.414) (Supplementary Table S4). Nevertheless, both variables exhibited significant interactions with deadwood presence: the positive effect of soil temperature was weaker beneath deadwood (estimate = -0.03, *p* = 0.002), and the relationship between soil moisture and DOC also differed between treatments (estimate = −0.01, *p* < 0.001). In addition, these effects varied with soil depth. However, as soil temperature and moisture were measured at 15 cm depth, depth-related differences in these relationships cannot be resolved explicitly and should be interpreted with caution. These findings indicate that deadwood modifies the relationship between microclimatic conditions and DOC.

**Fig. 2:**
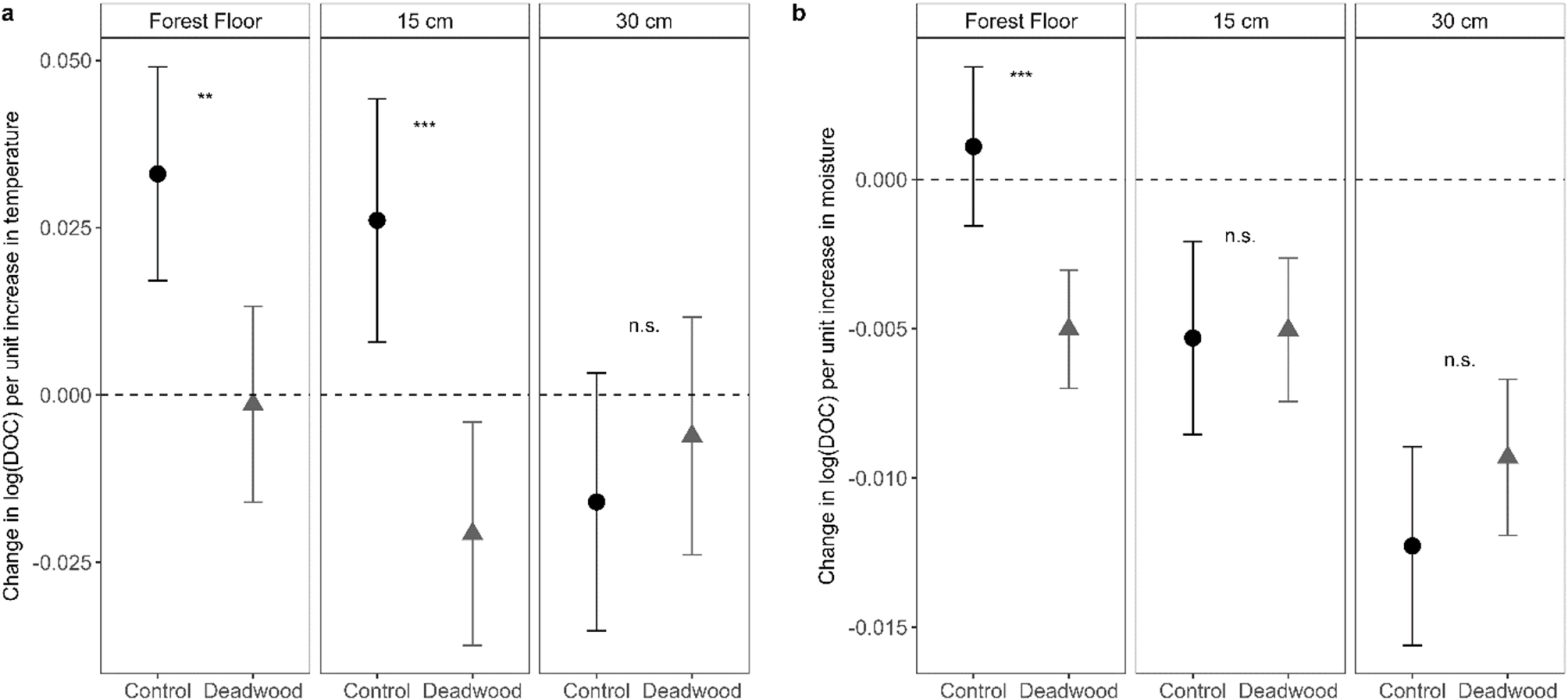
Effects of soil temperature and moisture on DOC concentrations under deadwood and control conditions. **a** Slopes describing the relationship between soil temperature and log-transformed DOC concentrations across treatments and soil depths. **b** Slopes describing the relationship between soil moisture and log-transformed DOC concentrations. Symbols represent the change in DOC per unit increase in temperature or moisture, error bars showing 95% confidence intervals. Positive values indicate increasing DOC, whereas negative values indicate decreasing DOC. Effects are considered significant when confidence intervals do not overlap zero. Asterisks indicate significant differences between treatments (n.s. = not significant).

The estimated effects (slopes) of soil temperature and moisture on DOC concentrations across treatments and soil depths are shown in Fig. 2. The symbols represent the estimated mean rate of change in DOC per unit increase in soil temperature or moisture, rather than absolute DOC concentrations. Positive slopes indicate an increase in DOC with rising temperature or moisture, whereas negative slopes indicate a decrease. Effects were considered statistically significant when the corresponding confidence intervals (error bars in Fig. 2) did not overlap zero.

In the forest floor, DOC concentrations increased significantly with temperature in the control (Fig. 2a), but not under deadwood, resulting in a significant difference of the temperature effect between treatments. At 15 cm depth, temperature had a significant positive effect on DOC in the control and a significant negative effect under deadwood, indicating opposing responses between treatments. At 30 cm depth, soil temperature had no significant effect on DOC in either treatment, and no differences between treatments were detected.

In the forest floor, soil moisture showed a positive but non-significant relationship with DOC in the control, while a significant negative effect was observed under deadwood (Fig. 2b). At 15 cm and 30 cm depth, soil moisture had a significant negative effect on DOC in both treatments.

Overall, the influence of soil temperature and moisture on DOC varied with both depth and treatment. Treatment-specific differences in temperature effects were most pronounced in the upper soil layers, whereas moisture effects became increasingly negative with depth.

## Discussion

### Deadwood effects on DOC differ with bedrock type and depth

We expected a greater increase in DOC concentrations beneath deadwood in silicate than in calcareous soils. This hypothesis was only partially confirmed. While DOC increased more strongly at 15 cm depth at the silicate site, this pattern was not consistent across depths. Overall, deadwood increased DOC concentrations at both sites, but the magnitude of this effect was strongly depth-dependent: lowest beneath the forest floor, highest at 15 cm, and smaller again at 30 cm depth.

DOC decreased more strongly with depth at the silicate site, likely due to higher sorption capacity associated with aluminum and iron oxides^47,48^ or limited bioturbation under acidic conditions^6^. The larger relative increase beneath deadwood at the silicate site may also reflect lower background DOC concentrations^28^.

These differences diminished with depth. At 30 cm, DOC dynamics appear increasingly controlled by sorption and transport rather than by DOC inputs. The convergence of DOC concentrations between deadwood and control plots suggests enhanced retention and processing in mineral soils, although underlying mechanisms cannot be resolved with the present data and require further investigation (e.g. analysis of the soil solid phase). Overall, site-specific soil properties strongly control DOC dynamics and the fate of C inputs from deadwood.

 The general increase in DOC beneath deadwood (across both bedrock types) agrees with previous studies^12–14,26–28^ and was detectable down to 30 cm depth, indicating transport into deeper soil layers. Possible mechanisms include additional inputs of dissolved organic matter from deadwood^14,16^ and enhanced microbial activity in the underlying soil^28,49^.

The weak effect beneath the forest floor likely reflects the dominance of litter-derived DOC, which can mask additional inputs from deadwood due to its large pool size and high DOC production. In mineral soils, background DOC concentrations are lower, which may increase the relative detectability of additional DOC inputs from deadwood. Accordingly, DOC concentrations were higher beneath deadwood than in control plots, particularly at 15 cm depth.

DOC in mineral soils (A horizon) is primarily leached from the forest floor rather than produced locally^16,50^, suggesting that differences between treatments are mainly driven by variation in DOC inputs rather than local production, and thus are unlikely to be explained by differences in DOC production between forest floor and mineral soil.

The decrease in DOC with depth in the mineral soil is consistent with earlier findings^16,51–54^ and can be explained by microbial processing, sorption to mineral surfaces, and occlusion within soil aggregates^6,51,55^. In addition to this vertical pattern, DOC concentrations were higher beneath deadwood than in control plots, particularly at 15 cm depth. This pronounced increase, particularly at the silicate site, suggests enhanced C turnover, potentially stimulated by additional DOC inputs from deadwood^21^.

At 30 cm depth, the difference between deadwood and control plots were smaller, likely reflecting reduced inputs of fresh dissolved organic matter and stronger sorption or microbial mineralization that control DOC dynamics and dominate over DOC sources^47,51,55,56^.

### Deadwood effects on DOC differ with tree species

We anticipated higher DOC concentrations beneath beech deadwood than beneath spruce under comparable site conditions, and our results confirmed this expectation. DOC increased substantially beneath beech, particularly at 15 cm depth, whereas no significant increase was observed beneath spruce, at least beneath the forest floor and at 15 cm depth. Species-specific differences were strongest at 15 cm depth and diminished towards the forest floor and 30 cm.

The pronounced increase beneath beech deadwood suggests strong species-specific controls via wood properties. Conifers and broadleaves differ in wood density, lignin composition, and C concentrations^14,57,58^, affecting decomposition and DOC release^14,58,59^. Higher DOC beneath beech likely reflects faster decomposition compared to spruce^59^. Changes in wood chemistry during decay – such as increasing lignin and decreasing cellulose contents – may further influence the release of soluble C^58^.

Microbial communities may also play a role in this species effect^14^. Beech and spruce are associated with distinct fungal decomposer communities: white-rot fungi, more prevalent in broadleaf species, degrade both lignin and cellulose, while brown-rot fungi, more common in conifers, primarily decompose cellulose and modify lignin^58^. These differences in fungal activity may influence both the quantity and quality of DOC released, potentially explaining the higher DOC concentrations observed beneath beech in this study.

As the logs of both species were in decay class 3, the observed differences are likely driven more by decomposition pathways than by decay stage. While log diameter can influence decay dynamics^59^, including it in the analysis did not improve model performance and does not explain the observed patterns. Overall, our results demonstrate that tree species strongly influences DOC dynamics beneath deadwood. This effect is driven by differences in wood chemistry, decomposition processes, and associated microbial communities, highlighting its importance in forest C cycling.

### Effects of soil temperature and moisture on DOC differ beneath deadwood

We hypothesized that deadwood exerts an influence on DOC dynamics through its control over the microclimate. While our results demonstrated that deadwood significantly affected soil temperature and moisture, the effect sizes were relatively small, with slightly lower soil temperature (−0.1 °C) and moisture (−2%) beneath deadwood. These findings suggest a limited microclimatic impact of deadwood under our study conditions.

Lower soil temperatures beneath deadwood have been reported previously^38,60–62^ mainly in open-canopy systems. In contrast, our closed-canopy sites likely reduced solar radiation and limited temperature extremes, thereby diminishing the potential for deadwood to exert strong additional microclimatic effects. Similarly, canopy regulation of water availability may explain the weak influence on soil moisture, despite known influence of deadwood on water storage and soil structure^6,63^.

Despite small microclimatic effects, relationships between soil temperature, moisture, and DOC differed between treatments, although effects remained weak. A positive relationship between soil temperature and DOC has been reported previously^64–67^, particularly beneath the forest floor^50,68^, reflecting increased microbial DOC production^69^. Such patterns may be especially apparent at the southwest-facing slope of the Tuttlingen site, where higher temperatures may enhance DOC production in the litter layer and subsequent transport into the soil, consistent with generally higher DOC concentrations observed in soil water at this slope. However, the positive relationship between temperature and DOC was absent beneath deadwood.

One possible explanation is the altered distribution of litter. When logs are oriented parallel to the slope, litter tends to accumulate above the log while less accumulates below it, where the suction cups were installed. Reduced litter input may limit DOC production and obscure temperature effects in the models. In control plots, the positive relationship between temperature and DOC likely reflects enhanced DOC production in the litter layer and increased leaching into the upper mineral soil under warmer conditions, an effect that is likely strongest in the topsoil layers. Differences in the temperature-DOC relationship between deadwood and control plots were most pronounced at 15 cm depth. While the relationship remained positive in control plots, it was negative beneath deadwood. Although weak, this pattern may indicate enhanced microbial consumption of DOC under deadwood, thereby reducing DOC concentrations (summarized by Kalbitz, et al.^47^).

At 30 cm depth, temperature-DOC relationships were negative in both deadwood and control plots, which may be attributed to a lower influence of forest floor leachates and higher decomposition rates at higher temperatures. However, temperature effects may be masked by water availability and soil properties in field studies^47^.

In contrast, soil moisture effects were more consistent across depths and treatments. DOC concentrations generally decreased with increasing moisture, in line with previous findings^50,54,70^. This pattern is likely attributable to dilution of DOC concentrations at high soil moisture levels. At the same time, drying and rewetting cycles can increase DOC concentrations (summarized by Kalbitz, et al.^47^). During dry periods, decomposition rates decrease and organic matter accumulates. After rewetting, this accumulated organic matter enters the soil solution, leading to increased DOC concentrations. However, this effect is likely short-lived and not captured by the suction cups used in our study. Alternative approaches, such as suction plates or grid lysimeters^71^, would allow direct tracking of drying and rewetting cycles and could be combined with DOC measurements.

As soil temperature and moisture were measured only at 15 cm depth and assumed to represent upper soil conditions, and given small effect sizes, results should be interpreted as trends rather than definitive conclusions. Variation in log size and slope position likely also influenced microclimate and DOC dynamics. However, including these variables did not increase explained variance.

## Conclusion

Deadwood can increase DOC concentrations in forest soils, but the magnitude of this effect varies with bedrock type, tree species, soil depth, and microclimatic conditions. In our study, DOC concentrations were significantly elevated beneath beech logs, particularly in the upper mineral soil, whereas spruce logs showed little influence. Soil temperature and moisture influenced DOC dynamics differently under deadwood and control plots. These results highlight that the contribution of deadwood to soil carbon inputs is context-dependent, and its persistence and fate in the soil is regulated by local soil conditions.

## Supplementary Information

Below is the link to the electronic supplementary material.

Supplementary Material 1

## Data Availability

The datasets generated during and/or analysed during the current study are available from the corresponding author on reasonable request.

## Abbreviations

C: Carbon
CO_2_: Carbon Dioxide
DOC: Dissolved Organic Carbon
POC: Particulate Organic Carbon
WRB: World Reference Base for Soil Resources
LOQ: Limit Of Quantification
LOD: Limit Of Detection
rAWC: Relative Available Water Capacity
FC: Field Capacity
PWP: Permanent Wilting Point
MAP: Mean Annual Precipitation
MAT: Mean Annual Temperature
DWD: Deutscher Wetterdienst
