# Supplementary material for: Deadwood effects on dissolved organic carbon in forest soils depend on bedrock type, tree species, and microclimate
Source: Sci Rep. 2026 Apr 28;16:13647. doi: 10.1038/s41598-026-50174-1 (PMC13125434; doi:10.1038/s41598-026-50174-1)
Supplement: Supplementary file 1 — Supplementary Material 1 [file 41598_2026_50174_MOESM1_ESM.pdf]

Deadwood effects on dissolved organic carbon in forest soils depend on bedrock type, tree species and microclimate

Lisa Rubin, Rebecca Nowack, Friederike Lang, Peter Stiasny, Heike Puhlmann

Table S1. Characteristics of sampled deadwood logs

Tree ID	Tree species	Length (m)	Diameter (cm)	Volume (m^3)	Decay class	Slope position
WAL BE A	*Fagus sylvatica*	5.75	18.5	0.15	4	Parallel
WAL BE B	*Fagus sylvatica*	9	19	0.26	3	Downslope
WAL BE C	*Fagus sylvatica*	9	14	0.14	4	Downslope
WAL SP A	*Picea abies*	11	27	0.63	3	Downslope
WAL SP B	*Picea abies*	4.21	14	0.06	3	Downslope
WAL SP C	*Picea abies*	6.5	15	0.11	4	Parallel
TUT NE A	*Fagus sylvatica*	7	20	0.22	3	Downslope
TUT NE B	*Fagus sylvatica*	2.55	11	0.02	4	Downslope
TUT NE C	*Fagus sylvatica*	3.7	15.5	0.07	4	Downslope
TUT SW A	*Fagus sylvatica*	4.55	30.75	0.34	3	Downslope
TUT SW B	*Fagus sylvatica*	4.3	24.5	0.20	4	Parallel
TUT SW C	*Fagus sylvatica*	11.3	27	0.65	3	Parallel

Table S2. Results of the linear mixed-effects model testing the effects of deadwood treatment, bedrock type, and soil depth on log-transformed dissolved organic carbon (DOC) concentrations. Estimates, standard errors (SE), 95% confidence intervals (CI), and p-values are shown for all fixed effects and their interactions. P-values were calculated using the Satterthwaite approximation. Test statistics (σ^2 = residual variance, τ_{00} = random intercept variance (between site), ICC = intraclass correlation coefficient) as well as the number of observations and marginal/conditional R^2 are displayed

	DOC (log-transformed)			
Predictors	*Estimate*	*Std. Error*	*95% CI*	*p-value*
(Intercept)	3.48	0.16	3.17 – 3.79	**<0.001**
Treatment [Deadwood]	0.25	0.05	0.14 – 0.36	**<0.001**
Bedrock [Silicate]	0.40	0.27	-0.13 – 0.94	0.136
Depth [15]	-1.35	0.06	-1.47 – -1.24	**<0.001**
Depth [30]	-1.63	0.06	-1.75 – -1.51	**<0.001**
Treatment [Deadwood] × Bedrock [Silicate]	-0.20	0.07	-0.35 – -0.06	**0.007**
Treatment [Deadwood] × Depth [15]	0.26	0.08	0.11 – 0.42	**0.001**
Treatment [Deadwood] × Depth [30]	0.16	0.09	-0.02 – 0.33	**0.077**
Bedrock [Silicate] × Depth [15]	-0.63	0.08	-0.79 – -0.48	**<0.001**
Bedrock [Silicate] × Depth [30]	-0.88	0.08	-1.04 – -0.72	**<0.001**
Treatment [Deadwood] × Bedrock [Silicate] × Depth [15]	0.33	0.11	0.11 – 0.55	**0.003**
Treatment [Deadwood] × Bedrock [Silicate] × Depth [30]	0.21	0.12	-0.02 – 0.44	**0.070**
Random Effects				
σ^2	0.24			
$\tau_{00\ Studysite}$	0.05			
ICC	0.16			
$N_{Studysite}$	3			
Observations	1722			
Marginal R^2 / Conditional R^2	0.741 / 0.783			

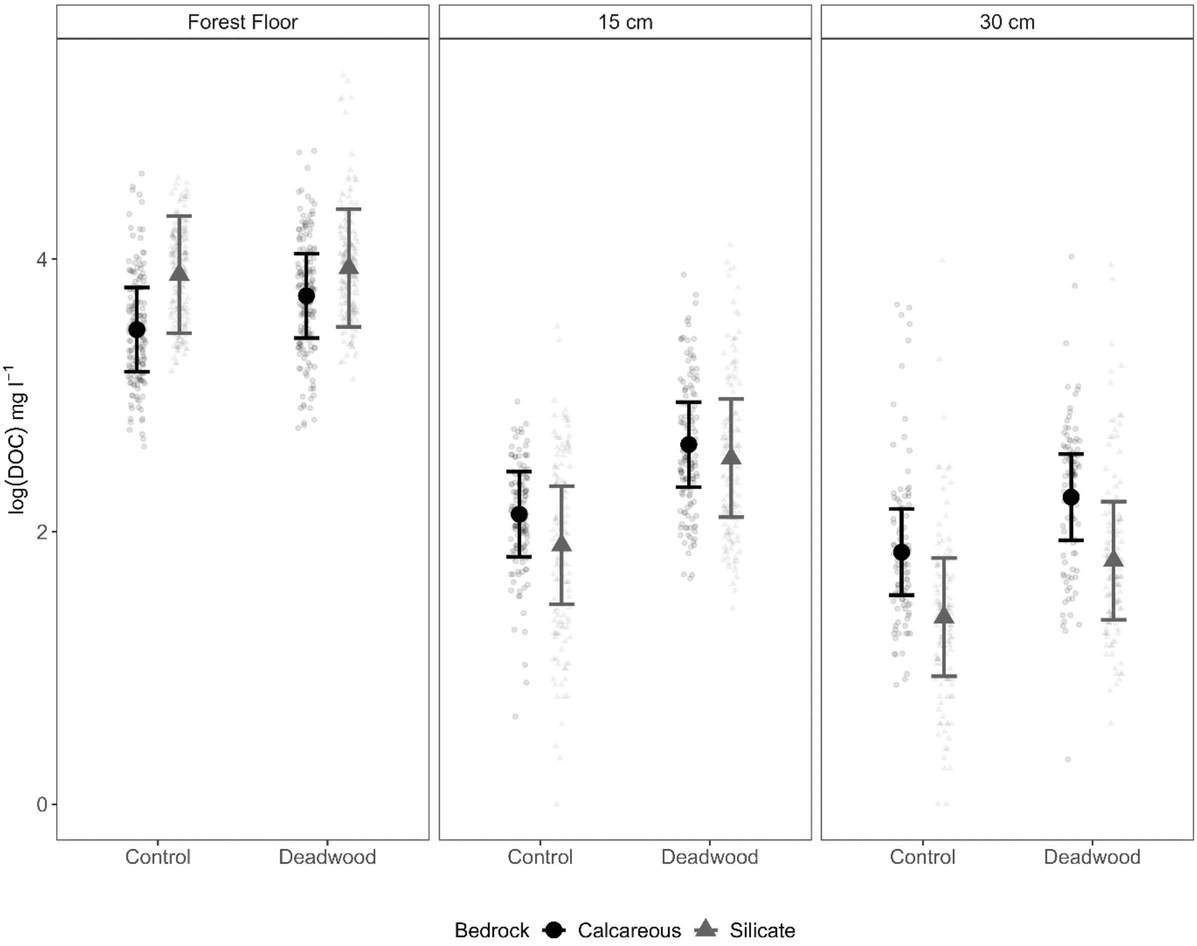

Fig. S1. Depth-dependent effects of deadwood and bedrock type on DOC concentrations in soil solution. Panels show log-transformed DOC concentrations under control and deadwood treatments for calcareous and silicate soils at different soil depths. Transparent points represent individual observations, while large symbols indicate estimated marginal means with error bars indicating 95 % confidence interval obtained from the linear mixed-effect model. Panels represent soil depth, and symbols distinguish between calcareous and silicate bedrock

Table S3. Results of the linear mixed-effects model testing the effects of deadwood treatment, tree species, and soil depth on log-transformed dissolved organic carbon (DOC) concentrations. Estimates, standard errors (SE), 95% confidence intervals (CI), and p-values are shown for all fixed effects and their interactions. P-values were calculated using the Satterthwaite approximation. Test statistics (σ^2 = residual variance, τ_{00} = random intercept variance (between Tree ID), ICC = intraclass correlation coefficient) as well as the number of observations and marginal/conditional R^2 are displayed

	DOC (log-transformed)			
Predictors	*Estimate*	*Std. Error*	*95% CI*	*p-value*
(Intercept)	3.83	0.15	3.53 – 4.14	**<0.001**
Treatment [Deadwood]	0.20	0.06	0.08 – 0.32	**0.001**
Species [Spruce]	0.10	0.22	-0.33 – 0.53	0.646
Depth [15]	-2.36	0.07	-2.49 – -2.22	**<0.001**
Depth [30]	-2.53	0.07	-2.67 – -2.39	**<0.001**
Treatment [Deadwood] × Species [Spruce]	-0.31	0.09	-0.47 – -0.14	**<0.001**
Treatment [Deadwood] × Depth [15]	1.40	0.10	1.21 – 1.60	**<0.001**
Treatment [Deadwood] × Depth [30]	0.48	0.10	0.28 – 0.68	**<0.001**
Species [Spruce] × Depth [15]	0.69	0.09	0.50 – 0.88	**<0.001**
Species [Spruce] × Depth [30]	0.04	0.10	-0.14 – 0.23	0.640
Treatment [Deadwood] × Species [Spruce] × Depth [15]	-1.43	0.13	-1.69 – -1.16	**<0.001**
Treatment [Deadwood] × Species [Spruce] × Depth [30]	-0.16	0.14	-0.42 – 0.11	0.248
Random Effects				
σ^2	0.18			
$\tau_{00\ Tree\_ID}$	0.07			
ICC	0.27			
N_{Tree_ID}	6			
Observations	926			
Marginal R^2 / Conditional R^2	0.823 / 0.871			

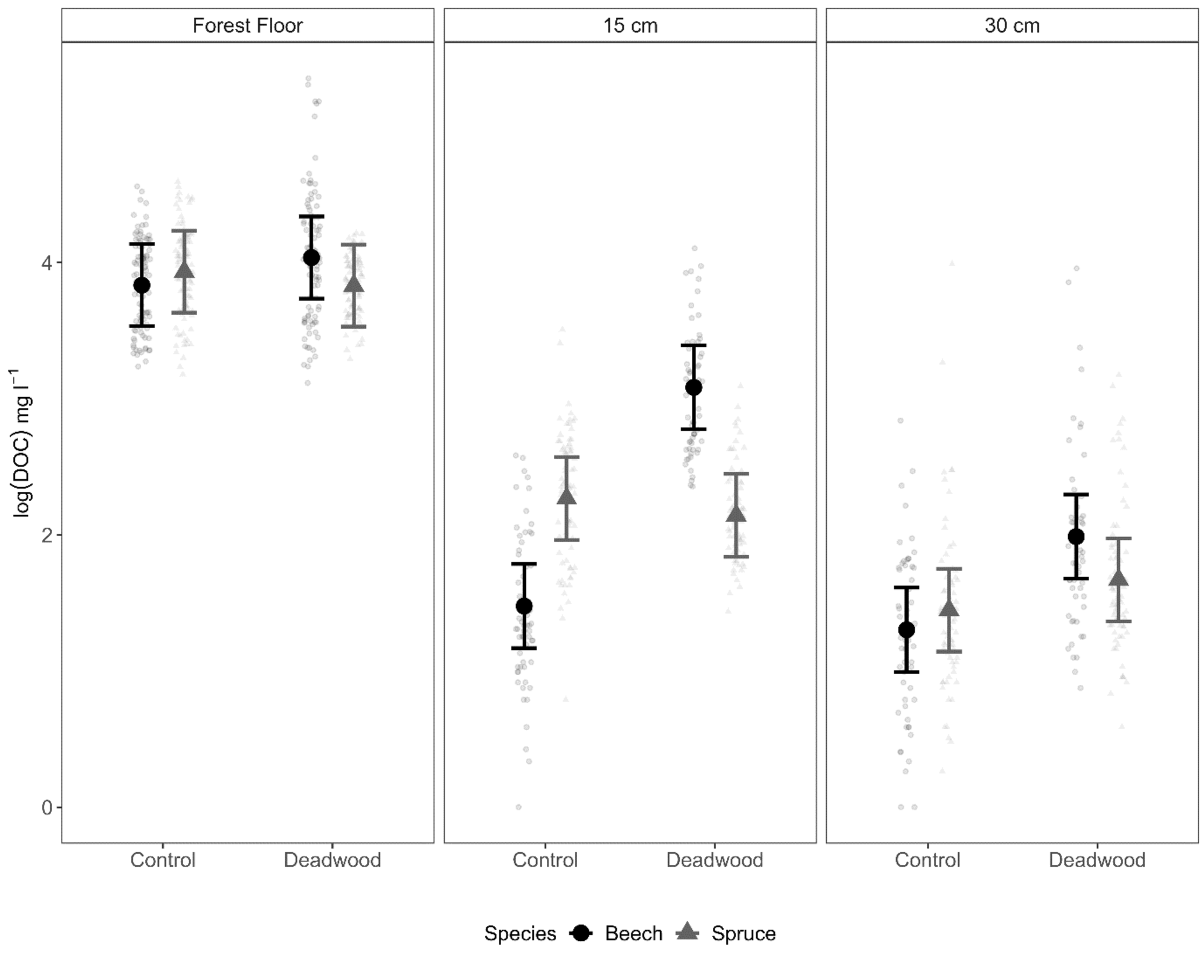

Fig. S2. Depth-dependent effects of deadwood and tree species on DOC concentrations in soil solution. Panels show log-transformed DOC concentrations under control and deadwood treatments under beech and spruce at different soil depths. Transparent points represent individual observations, while large symbols indicate estimated marginal means with error bars indicating 95 % confidence interval obtained from the linear mixed-effect model. Panels represent soil depth, and symbols distinguish between beech and spruce deadwood

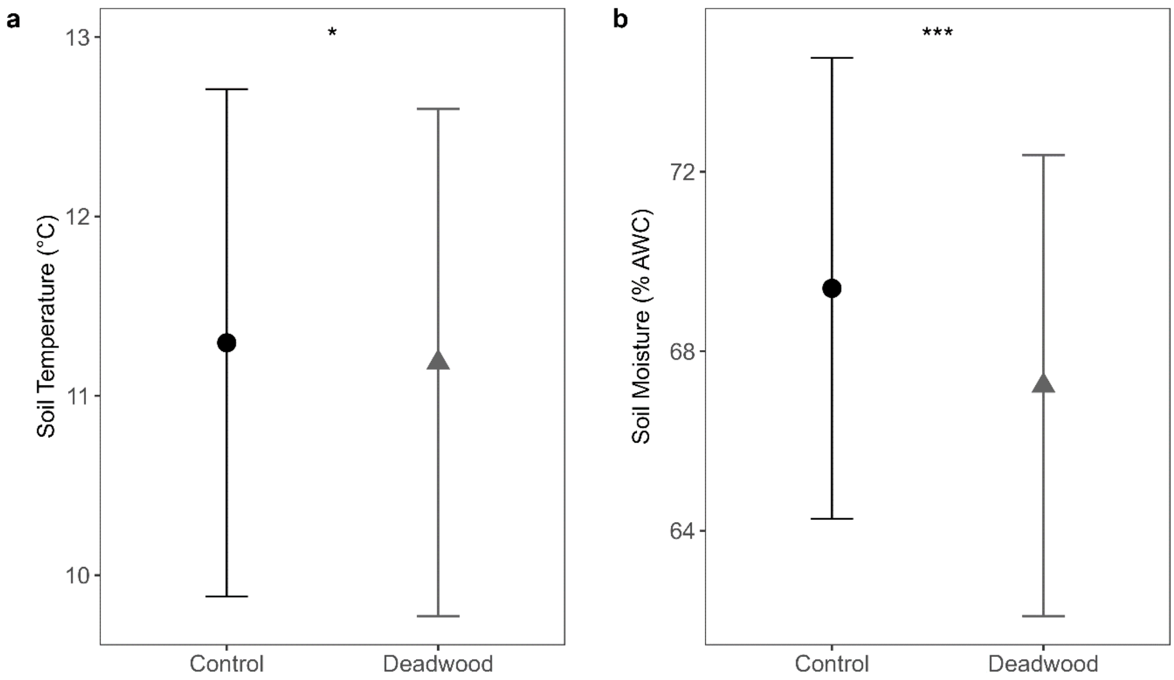

Fig. S3. Effects of deadwood presence on (a) soil temperature and (b) soil moisture (% AWC) at 15 cm depth. Symbols represent estimated marginal means from linear mixed-effects models, with error bars indicating 95 % confidence intervals. Asterisks denote significant differences between deadwood and control

Table S4. Results of the linear mixed-effects model testing the effects of deadwood treatment, soil temperature, soil moisture, and soil depth on log-transformed dissolved organic carbon (DOC) concentrations. Estimates, standard errors (SE), 95% confidence intervals (CI), and p-values are shown for all fixed effects and their interactions. P-values were calculated using the Satterthwaite approximation. Test statistics (σ^2 = residual variance, τ_{00} = random intercept variance (between site), ICC = intraclass correlation coefficient) as well as the number of observations and marginal/conditional R^2 are displayed

	DOC (log-transformed)			
Predictors	*Estimate*	*Std. Error*	*95% CI*	*p-value*
(Intercept)	3.67	0.09	3.49 – 3.84	**<0.001**
Treatment [Deadwood]	0.18	0.05	0.09 – 0.27	**<0.001**
Soil Temperature	0.03	0.01	0.02 – 0.05	**<0.001**
Soil Moisture	0.00	0.00	-0.00 – 0.00	0.414
Depth [15]	-1.62	0.05	-1.72 – -1.52	**<0.001**
Depth [30]	-2.06	0.05	-2.16 – -1.96	**<0.001**
Treatment [Deadwood] × Soil Temperature	-0.03	0.01	-0.06 – -0.01	**0.002**
Treatment [Deadwood] × Soil Moisture	-0.01	0.00	-0.01 – -0.00	**<0.001**
Soil Temperature × Soil Moisture	-0.00	0.00	-0.00 – 0.00	0.303
Treatment [Deadwood] × Depth [15]	0.35	0.07	0.22 – 0.49	**<0.001**
Treatment [Deadwood] × Depth [30]	0.24	0.07	0.11 – 0.38	**0.001**
Soil Temperature × Depth [15]	-0.01	0.01	-0.03 – 0.02	0.572
Soil Temperature × Depth [30]	-0.05	0.01	-0.07 – -0.02	**<0.001**
Soil Moisture × Depth [15]	-0.01	0.00	-0.01 – -0.00	**0.002**
Soil Moisture × Depth [30]	-0.01	0.00	-0.02 – -0.01	**<0.001**
Treatment [Deadwood] × Soil Temperature × Soil Moisture	0.00	0.00	-0.00 – 0.00	**0.077**
Treatment [Deadwood] × Soil Temperature × Depth [15]	-0.01	0.02	-0.05 – 0.02	0.454
Treatment [Deadwood] × Soil Temperature × Depth [30]	0.04	0.02	0.01 – 0.08	**0.010**
Treatment [Deadwood] × Soil Moisture × Depth [15]	0.01	0.00	0.00 – 0.01	**0.016**
Treatment [Deadwood] × Soil Moisture × Depth [30]	0.01	0.00	0.00 – 0.01	**0.001**
Soil Temperature × Soil Moisture × Depth [15]	0.00	0.00	-0.00 – 0.00	0.345
Soil Temperature × Soil Moisture × Depth [30]	0.00	0.00	-0.00 – 0.00	0.682
Treatment [Deadwood] × Soil Temperature × Soil Moisture × Depth [15]	-0.00	0.00	-0.00 – 0.00	0.148
Treatment [Deadwood] × Soil Temperature × Soil Moisture × Depth [30]	-0.00	0.00	-0.00 – 0.00	0.677
Random Effects				
σ^2	0.23			
$\tau_{00\ Studysite}$	0.02			
ICC	0.08			
$N_{Studysite}$	3			
Observations	1632			
Marginal R^2 / Conditional R^2	0.770 / 0.790			
